# Supplementary figures and images for: Lipid packing contributes to the confinement of caveolae to the plasma membrane
Source: eLife. 2026 Jul 6;14:RP108369. doi: 10.7554/eLife.108369 (PMC13336769; doi:10.7554/eLife.108369)

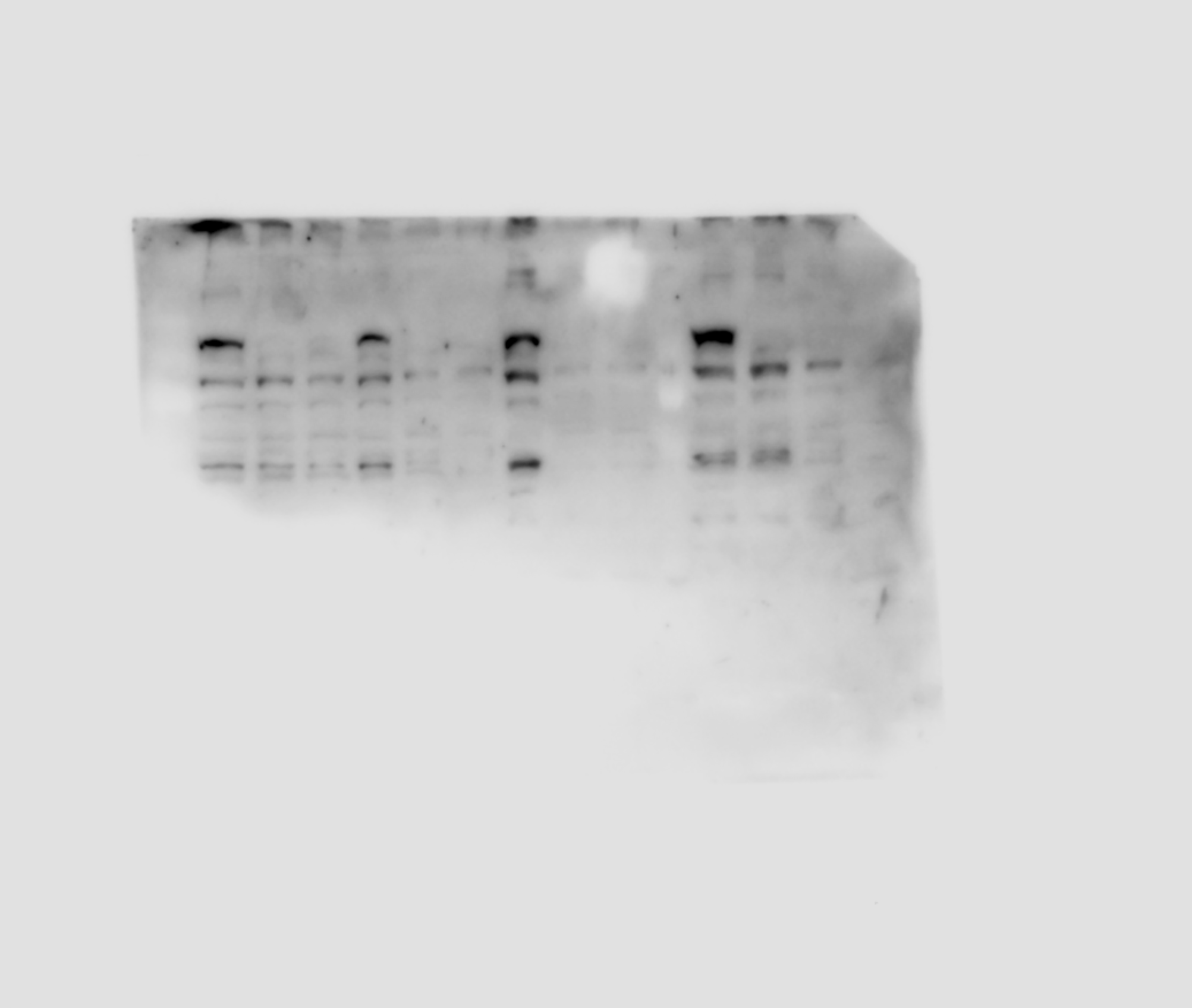

Supplement: Figure 1—source data 1. [file elife-108369-fig1-data1.zip › Raw WB files/DynTKO_aDyn2.tif]

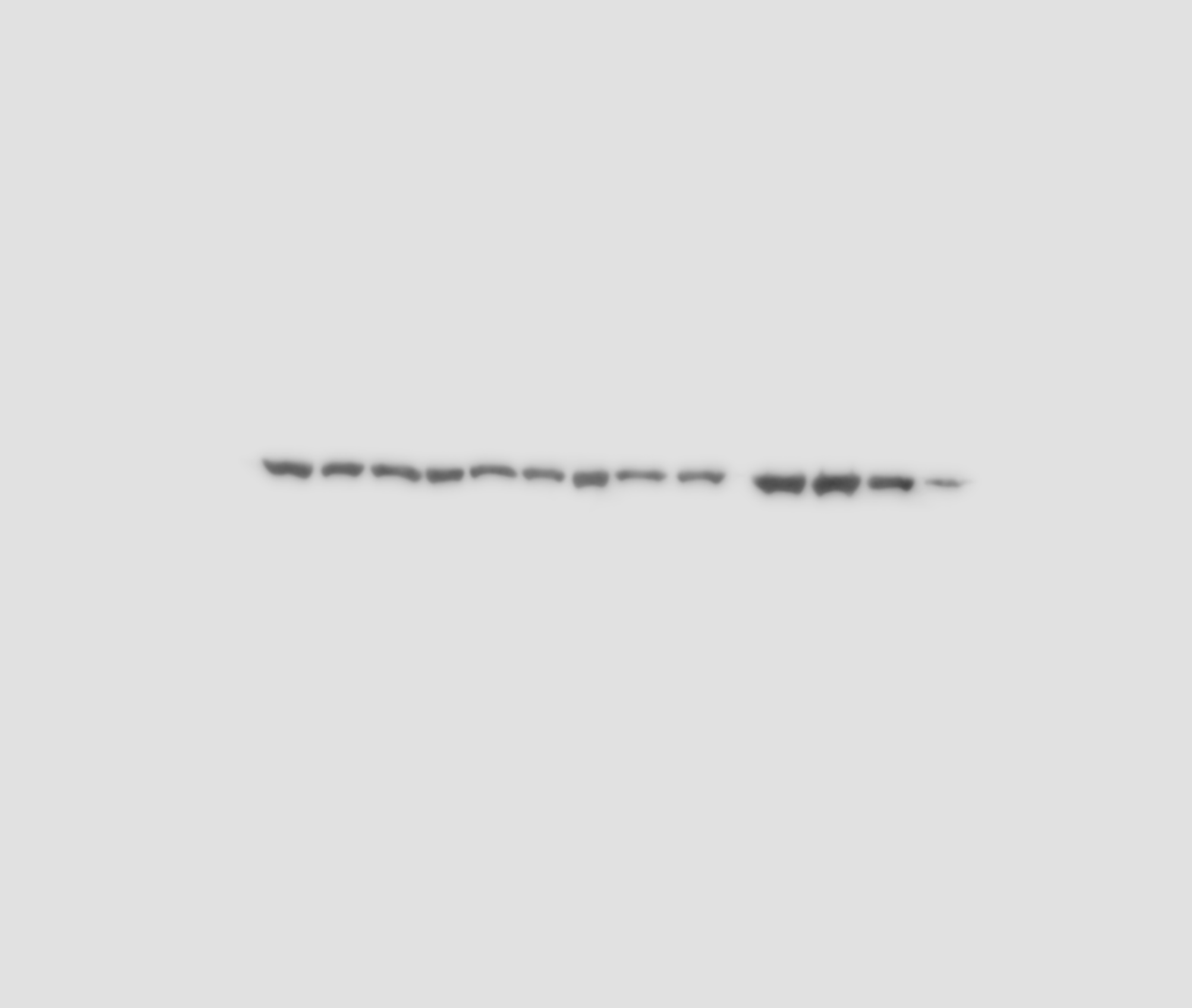

Supplement: Figure 1—source data 1. [file elife-108369-fig1-data1.zip › Raw WB files/DynTKO_actin.tif]

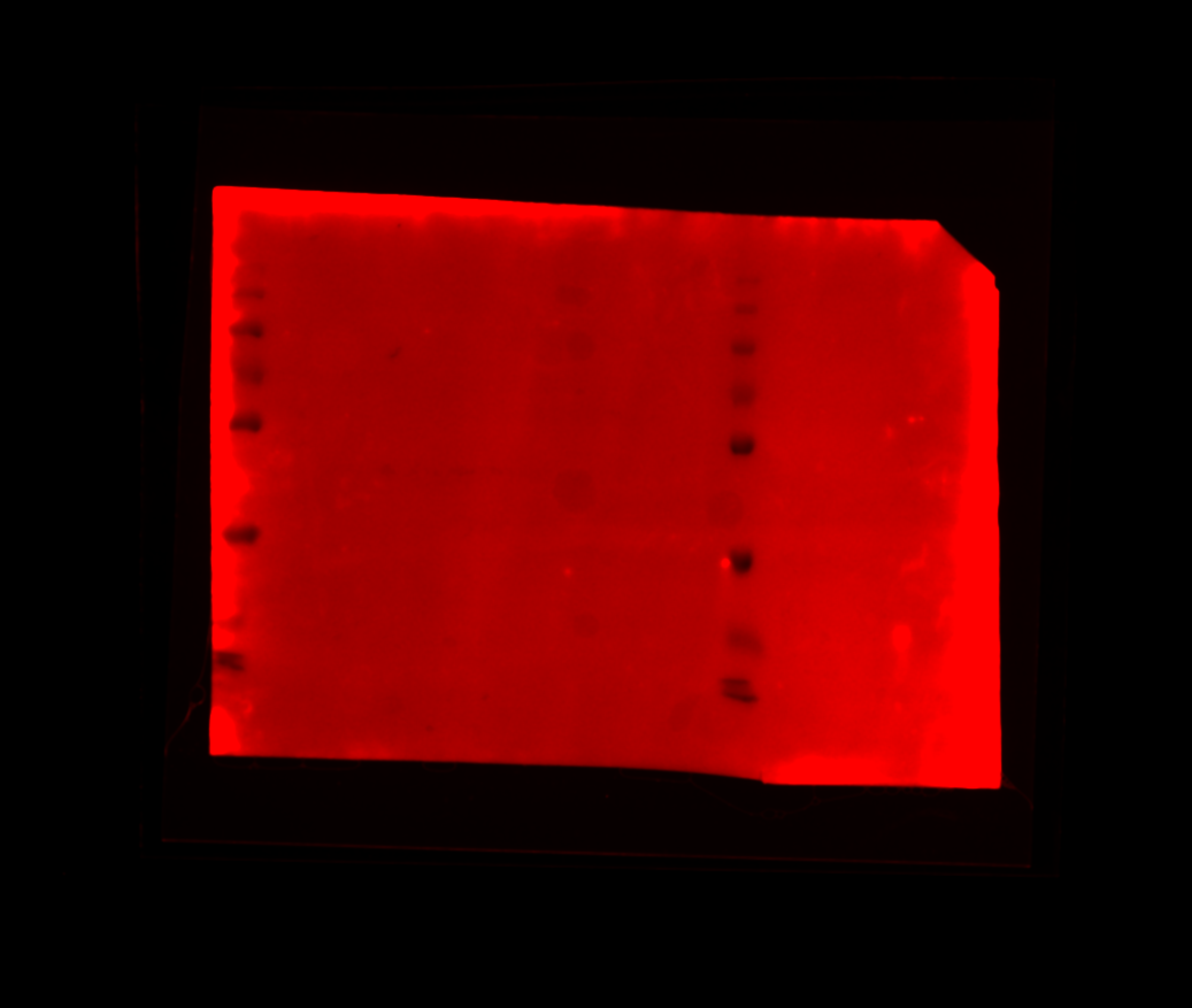

Supplement: Figure 1—source data 1. [file elife-108369-fig1-data1.zip › Raw WB files/DynTKO_ladder.tif]
