# Supplementary material for: Lipid packing contributes to the confinement of caveolae to the plasma membrane
Source: eLife. 2026 Jul 6;14:RP108369. doi: 10.7554/eLife.108369 (PMC13336769; doi:10.7554/eLife.108369)
Supplement: Peer review history

# Editor report: eLife Assessment

**DOI:** 10.7554/eLife.108369.3.sa0

**Authors:** Yuichi Wakana

**Affiliations:** Tokyo University of Pharmacy and Life Sciences Japan

**Keywords:** Convincing, Solid, Important

On the basis of **convincing** computational, biophysical, and cell-based evidence, this study reports the **important** finding that the dynamin inhibitor Dyngo-4a broadly affects lipid packing and plasma membrane dynamics, independently of its action on dynamin. The evidence, obtained by a wide range of methods including a newly developed assay visualizing internalized caveolae, provides **solid** support for the authors' main claim on the role of lipid packing in caveolae internalization. This work will be of significant interest to cell biologists, biophysicists, and chemists interested in membrane remodeling and drug-membrane interactions.

[Editors' note: this paper was reviewed by Review Commons.]

# Referee report: Reviewer #1 (Public review)

**DOI:** 10.7554/eLife.108369.3.sa1

Summary:

The authors use Dyngo-4a, a known Dynamin inhibitor to test its influence on caveolar assembly and surface mobility. They investigate whether it incorporates into membranes with Quartz-Crystal Microbalance, they investigate how it is organized in membranes using simulations. Finally, they use lipid-packing sensitive dyes to investigate lipid packing in the presence of Dyngo-4a, membrane stiffness using AFM and membrane undulation using fluorescence microscopy. They also use a measure they call "caveola duration time" to claim that something happens to caveolae after Dyngo-4a addition and using this parameter, they do indeed see an increase in it in response to Dyngo-4a, which is reduced back to the baseline after addition of cholesterol.

Overall, the authors claim: (1) Dyngo-4a inserts into the membrane and this (2) results in "a dramatic dynamin-independent inhibition of caveola scission". (3) Dyngo-4a was inserted and positioned at the level of cholesterol in the bilayer and (4) Dyngo-4a-treatment resulted in decreased lipid packing in the outer leaflet of the plasma membrane (5) but Dyngo-4a did not affect caveola morphology, caveolae-associated proteins, or the overall membrane stiffness (6) acute addition of cholesterol counteracts the block in caveola scission caused by Dyngo-4a.

Overall, in this reviewers opinion, after the additional experiments in the review process, all claims are now well-supported by the presented data from electron and live cell microscopy, QCM-D and AFM.

Significance:

A number of small molecule inhibitors for the GTPase dynamics exist, that are commonly used tools in the investigation of endocytosis. This goes as far that the use of some of these inhibitors alone is considered in some publications as sufficient to declare a process to be dynamin-dependent. However, this is not always correct, as there are considerable off-target effects, including the inhibition of caveolar internalization by a dynamin-independent mechanism. This is important, as for example the influence of dynamin small molecule inhibitors on chemotherapy resistance is currently investigated (see for example Tremblay et al., Nature Communications, 2020).

The investigation of the true effect of small molecules discovered as and used as specific inhibitors and their offside effects is extremely important and this reviewer applauds the effort. It is important that inhibitors are not used alone, but other means of targeting a mechanism are exploited as well in functional studies. The audience here thus is besides membrane biophysicists interested in the immediate effect of the small molecule Dyngo-4a also cell biologists and everyone using dynamic inhibitors to investigate cellular function.

Comments on revised version.

Overall, in this reviewer's opinion, after the additional experiments in the review process, all claims are now well-supported by the presented data from electron and live cell microscopy, QCM-D and AFM.

# Referee report: Reviewer #2 (Public review)

**DOI:** 10.7554/eLife.108369.3.sa2

Summary:

In this manuscript, the authors probe the mechanisms by which Dyngo-4a, a dynamin inhibitor used to block endocytosis, impact caveolae dynamics. They provide compelling evidence that Dyngo-4a inhibits caveolae dynamics and endocytosis (as well as several other aspects of plasma membrane dynamics) by a dynamin-independent mechanism. They also provide strong computational and experimental data showing that Dyngo-4a inserts into membranes and decreases lipid packing in the outer leaflet of the plasma membrane. Finally, they demonstrate that the addition of excess cholesterol to cells reverses the effects of Dyngo-4a on caveolae dynamics, presumably by reversing lipid packing defects. Based on these findings they conclude that lipid packing regulates caveolae dynamics and endocytosis in a cholesterol-dependent manner.

This work should be of value to cell biologists interested in plasma membrane remodeling and membrane trafficking, biophysicists that study small molecule/membrane interactions and membrane remodeling processes, and chemists interested in designing drugs to target membrane trafficking machinery and pathways.

Strengths and weaknesses:

This work addresses the important topic of how a widely used endocytic inhibitor actually works. In the process of addressing this question, the authors uncover unexpected connections between how lipids are packed in cell membranes and membrane dynamics. The methods are appropriate and many of the claims made in this work are well supported by data.

The authors have also been responsive to comments raised during review by including additional experimental evidence that Dyngo-4a inhibits caveolae endocytosis as well as documenting the effects of Dyngo-4a on caveolae morphology.

The work also raises some interesting questions for the future. As one example, the authors note that in addition to inhibiting caveolar dynamics, Dyngo-4a inhibits generalized plasma membrane mobility, transferrin uptake, and fusion of fusogenic liposomes to the plasma membrane. More work will be required to determine whether these events are mediated by a common, lipid packing-dependent mechanism.

# Author comment: Author response

**DOI:** 10.7554/eLife.108369.3.sa3

**Authors:** Elin Larsson, Aleksei Kabedev, Hudson Pace, Jakob Lindwall, Fouzia Bano, James Rae, Robert G Parton, Christel A Bergström, Ingela Parmryd, Marta Bally, Richard Lundmark

**Affiliations:** Umeå University Umeå Sweden; Uppsala University Uppsala Sweden; Umeå University Umeå Sweden; Umeå University Umeå Sweden; Umeå Universitet Umeå Sweden; The University of Queensland Brisbane Australia; The University of Queensland Brisbane Australia; Uppsala University Uppsala Sweden; University of Gothenburg Göteborg Sweden; Clinical Microbiology, Umeå University Umeå Sweden; Umeå University Umeå Sweden

The following is the authors’ response to the original reviews

**eLife Assessment:**

This study reports the important finding that the dynamin inhibitor Dyngo-4a broadly affects lipid packing and plasma membrane dynamics, independently of its action on dynamin. While solid computational, biophysical, and cell-based evidence supports this conclusion, there is incomplete support for the authors' main claim on the role of lipid packing in caveolae internalization, as the causal relationship remains unclear and direct analyses are lacking. With stronger evidence, this work would be of significant interest to cell biologists, biophysicists, and chemists interested in membrane remodeling and drug-membrane interactions.

We are thankful for the very positive feedback and enthusiasm for our work and sincerely thank all the reviewers for their time, their constructive criticism and valuable comments. Based on this, we have revised our manuscript as detailed below in the point-by-point response where the responses to reviewers’ comments are indicated in blue font. Text edits in the revised manuscript are indicated in red font.

We agree that providing sufficient evidence for inhibition of caveolae endocytosis by Dyngo-4a is critical and have therefore worked hard on identifying suitable assays that enable conclusive experiments as described below. We have now added a new figure with data that we think firmly supports our statement that caveolae internalization is restricted by Dyngo-4a. Additionally, EM images and quantifications of caveola morphology with or without treatment has been added within the same figure. Taken together, we believe that we have provided strong data to support this main claim and challenged this hypothesis as far as current methodology allows. Therefore, we hope that the revised manuscript warrants a new eLife assessment and we would like this to be the version of accord for the publication in eLife.

Point-by-point response to reviewers comments

**Reviewer #1 (Public review):**

The authors use Dyngo-4a, a known Dynamin inhibitor to test its influence on caveolar assembly and surface mobility. They investigate whether it incorporates into membranes with Quartz-Crystal Microbalance, they investigate how it is organized in membranes using simulations. Finally, they use lipid-packing sensitive dyes to investigate lipid packing in the presence of Dyngo-4a, membrane stiffness using AFM and membrane undulation using fluorescence microscopy. They also use a measure they call "caveola duration time" to claim that something happens to caveolae after Dyngo-4a addition and using this parameter, they do indeed see an increase in it in response to Dyngo-4a, which is reduced back to the baseline after addition of cholesterol.

Overall, the authors claim: (1) Dyngo-4a inserts into the membrane and this (2) results in "a dramatic dynamin-independent inhibition of caveola scission". (3) Dyngo-4a was inserted and positioned at the level of cholesterol in the bilayer and (4) Dyngo-4a-treatment resulted in decreased lipid packing in the outer leaflet of the plasma membrane (5) but Dyngo-4a did not affect caveola morphology, caveolae-associated proteins, or the overall membrane stiffness (6) acute addition of cholesterol counteracts the block in caveola scission caused by Dyngo-4a.

Overall, in this reviewers opinion, claims 1, 3, 4, 5 are well-supported by the presented data from electron and live cell microscopy, QCM-D and AFM.

We thank the reviewer for these positive and encouraging words and believe that the new experiments added to the manuscript has provided strong evidence that caveola internalization is greatly inhibited by Dyngo-4a (see below).

However, there is no convincing assay for caveolar endocytosis presented besides the "caveola duration" which although unclearly described seems to be the time it takes in imaging until a caveolae is not picked up by the tracking software anymore in TIRF microscopy. Since the main claim of the paper is a mechanism of caveolar endocytosis being blocked by Dyngo-4a, a true caveolar internalization assay is required to make this claim. This means either the intracellular detection of not surface connected caveolar cargo or the quantification of caveolar movement from TIRF into epifluorescence detection in the fluorescence microscope. Otherwise, the authors could remove the claim and just claim that caveolar mobility is influenced.

We thank the reviewer and agree that this is a very important point to verify. Therefore, we have worked hard to quantify the endocytosis of caveolae in thin sections of MEF cells using transmission electron microscopy. By incubating cells with externally added HRP for two-minutes followed by washing, vesicles internalized during this period can be contrasted and distinguished from surface associated vesicles. Sections were quantified by counting both surface-associated and internalized caveolae and CCVs (see figure below). Surface associated caveolae and CCVs can be distinguished based on size and shape for CCV the presence of a coat, but the number of vesicles per image is very low because a cross section has to go right through the vesicle. Furthermore, although internalized caveolae and CCVs can be differentiated by size, it is much harder to separate these from other vesicles, tubules and tubular endosomes positive for HRP. We detect an approximate 50% reduction in internalized caveolae and CCVs (ie. containing the internalized marker) in Dyngo-4a cells, which confirms that internalization is impaired following Dyngo-4a treatment. Yet, CCV endocytosis was simultaneously confirmed by Tfn uptake assay to be reduced by a greater extent, approximately 95%. We believe that this discrepancy in numbers is due to the low frequency of counted vesicles per section and the difficulties in distinguishing different internalized vesicles and endosomal tubules making a robust quantification of endocytic events difficult. It is also important to note that the EM assay relies on structural criteria to identify only the budded CCVs and caveolae containing the internalized marker, in transit to the early endosome. Other labeled structures are excluded. In contrast, uptake of Tfn into endosomes would also be measured by the light microscopy assay. Therefore, we have chosen not to include these data in the revised manuscript.

**Author response image 1.** 

Instead, we have developed a new assay in which we can quantify internalization in whole cells and clearly separate internalized caveolae from those that are surface associated or have fused with endosomal structures. For this we use the HeLa FlpIn Cav1-GFP cells which are induced to express Cav1-GFP at endogenous levels to label caveolae. The cells are incubated for five minutes with fluorescent CTxB known to be internalized by caveolae (but also via other mechanisms). To be able to separate internalized caveolae from early endosomes, cells were fixed and labelled with antibodies against the marker EEA1. Cells were analyzed by fluorescence microscopy and confocal z-stacks of entire cells were recorded. The data was analyzed by software to identify only the caveolae that were positive for CTxB but negative for EEA1. The results from quantification showed a very clear inhibition in the number of internalized caveolae in Dyngo-4a treated cells in comparison to control cells. These data have been included in the manuscript as an important new figure 2 together with TEM data where we quantify the morphology of surface associated caveolae with or without Dyngo-4a treatment. We have also extensively edited the text in the results section to describe these new data and to convey that Dyngo-4a indeed affects internalization. We are very happy to have established means to address this important point by extending the current methodology and tools. Together with the TIRF data and FRAP data we believe that we have provided strong data for this claim and challenged our hypothesis as far as current methodology allows.

Significance:

A number of small molecule inhibitors for the GTPase dynamics exist, that are commonly used tools in the investigation of endocytosis. This goes as far that the use of some of these inhibitors alone is considered in some publications as sufficient to declare a process to be dynamin-dependent. However, this is not correct, as there are considerable off-target effects, including the inhibition of caveolar internalization by a dynamin-independent mechanism. This is important, as for example the influence of dynamin small molecule inhibitors on chemotherapy resistance is currently investigated (see for example Tremblay et al., Nature Communications, 2020). The investigation of the true effect of small molecules discovered as and used as specific inhibitors and their offside effects is extremely important and this reviewer applauds the effort. It is important that inhibitors are not used alone, but other means of targeting a mechanism are exploited as well in functional studies. The audience here thus is besides membrane biophysicists interested in the immediate effect of the small molecule Dyngo-4a also cell biologists and everyone using dynamic inhibitors to investigate cellular function.

Thank you for the comments. We very much appreciate the interest and enthusiasm of the reviewer for our work. This has inspired and supported us to perform additional work for the revision of our manuscript.

**Reviewer #2 (Public review):**

In this manuscript, the authors probe the mechanisms by which Dyngo-4a, a dynamin inhibitor used to block endocytosis, disrupts caveolae dynamics. They provide compelling evidence that Dyngo-4a inhibits caveolae dynamics and endocytosis (as well as several other aspects of plasma membrane dynamics) by a dynamin-independent mechanism. They also provide strong computational and experimental data showing that Dyngo-4a inserts into membranes and decreases lipid packing in the outer leaflet of the plasma membrane. Finally, they demonstrate that the addition of excess cholesterol to cells reverses the effects of Dyngo-4a on caveolae dynamics, presumably by reversing lipid packing defects. Based on these findings they conclude that lipid packing regulates caveolae dynamics and endocytosis in a cholesterol-dependent manner.

This work should be of value to cell biologists interested in plasma membrane remodeling and membrane trafficking, biophysicists that study small molecule/membrane interactions and membrane remodeling processes, and chemists interested in designing drugs to target membrane trafficking machinery and pathways.

This work addresses the important topic of how a widely used endocytic inhibitor actually works. In the process of addressing this question, the authors uncover unexpected connections between how lipids are packed in cell membranes and membrane dynamics. The methods are appropriate and many of the claims made in this work are well supported by data.

We very much appreciate the thorough review and very positive feedback constructive critique and thank the reviewer for the time spent on our manuscript.

Weaknesses:

I appreciate that the manuscript has already gone through one round of revisions and that many of the concerns from the previous reviewers appear to have been addressed. However, as an interested reader, I would like to offer several additional comments for the authors to consider.

(1) It is not clear based on the data presented whether the effects of Dyngo-4a on lipid packing give rise to defects in caveolae dynamics or if these effects are merely correlated. To show this more definitively, one might expect additional experimental approaches to be used to perturb lipid packing. I appreciate this is probably beyond the scope of the current study. However, it seems important for the manuscript to be clear about how far this interpretation can be pushed in the absence of additional independent lines of evidence.

We are very proud of the direct experimental support of the effect on lipid packing that we have performed using incorporation of extra cholesterol to the membrane which supports these effects are not merely correlated. Unfortunately, specifically perturbing lipid packing in other ways and conclusively interpreting such data is not uncomplicated. We agree that data and conclusions should be further challenged but we believe that this goes beyond the scope of this manuscript.

(2) On a related note, it is not obvious how changes in lipid packing in the outer leaflet could impact caveolae dynamics. It would be helpful to include a cartoon illustrating how this might work.

Thank you for pointing out this important aspect. We have elaborated on this within the discussion and referred to our recently published perspective article in Nature Cell Biology ('A lipid-centric view of endocytosis by caveolae' Parton, Kozlov and Lundmark DOI: 10.1038/s41556-026-01945-5) where this topic is extensively discussed. In short, insertion of the 8S disc in the inner leaflet of the PM replaces approximately 250 lipids and spans the entire thickness of the leaflet. The insertion of the flat, hydrophobic phase of the 8S disc, that faces the outer leaflet, results in a differential contact energy favoring the uneven packing of lipids and preferred accumulation of cholesterol in the PM of mammalian cells. Increased cholesterol content in the PM leads to more tilt and splay and hence curvature generation and, if not constrained by EHD2, scission. Thus, the distinct lipid packing of cholesterol and sphingomyelin opposite the Cav1 complex is key to drive curvature generation and internalization of caveolae.

We agree that a schematic figure could be nice to illustrate how packing affects caveolae internalization. However, we realized that providing a comprehensible concept this would require an extensive figure with vast discussions in the text. Therefore, we have chosen not to include this here, but refer to the figures in Parton et al. Nature Cell Biology DOI: 10.1038/s41556-026-01945-5

(3) The authors note that Dyngo-4a inhibits several dynamic processes including generalized plasma membrane mobility (Fig 4A&B), transferrin uptake (Fig S4C), and fusion of fusogenic liposomes (Fig S4G). This clearly indicates there is a major disruption of the plasma membrane going on here that is not limited to caveolae. They go on to show that the addition of cholesterol reverses the effects of Dyngo-4a on caveolae dynamics. However, they do not discuss whether adding back cholesterol has similar effects on plasma membrane mobility and transferrin uptake. This information could help to further pinpoint whether the mechanisms of action are shared, and if the role of cholesterol is more general in controlling these events or is instead specific to caveolae.

Yes, this is correct, and we agree that this important finding leads to many follow up questions on the mechanism of action of Dyngo-4a on cellular processes. Yet, to dissect the mechanism for all these processes goes way beyond the scope and our resources for this manuscript.

(4) In Fig 4C, the morphology of the neck region of the Dyngo-4a treated caveolae structure appears to be "pinched" compared to the control. I appreciate that more EM studies are underway. It would be useful to specifically compare the morphology of the caveolae as part of those studies.

Thanks, this is a relevant and interesting question. In the revised manuscript, we have therefore performed and included extra quantitative EM data addressing the morphology of caveolae. Based on this we conclude that there is no statistically significant difference in the height, width or neck diameter of caveolae treated with Dyngo-4a in comparison to control cells. When analyzing the ratio of height, width and neck diameter of each caveolae, there is a trend in that neck diameter is increased in Dyngo-4a-treated cells. These data have been included in the new figure 2 A-B and discussed in the text.

(5) In Line 91, a statement is made that 8S complex formation requires cholesterol. This is debatable, as they appear to form in *E. coli* in the absence of cholesterol (reference 14).

Thank you, we have clarified that this statement is referring to mammalian cells.

Some minor spelling errors include:

Line 66 generrating

Line 182 signigicantly

Line 197 treatmend

Line 347 succefully

These errors have been corrected
